# Human–Animal Interaction and Older Adults: An Overview

**DOI:** 10.3389/fpsyg.2017.01416

**Published:** 2017-08-21

**Authors:** Nancy R. Gee, Megan K. Mueller, Angela L. Curl

**Affiliations:** ^1^Department of Psychology, State University of New York, Fredonia NY, United States; ^2^WALTHAM Centre for Pet Nutrition Leicestershire, United Kingdom; ^3^Tufts Institute for Human-Animal Interaction, Department of Clinical Sciences, Cummings School of Veterinary Medicine at Tufts University, North Grafton MA, United States; ^4^Jonathan M. Tisch College of Civic Life at Tufts University, Medford MA, United States; ^5^Family Science and Social Work and Scripps Gerontology Center, Miami University, Oxford OH, United States

**Keywords:** human–animal interaction, pet ownership, animal-assisted therapy, aging, older adults

## Abstract

Both pet ownership and animal-assisted therapy are becoming increasingly popular in the United States, and the science of human–animal interaction (HAI) seeks to explore how these relationships with animals can impact health and well-being. In particular, one burgeoning area of research is the role of HAI in healthy aging, given the potential for HAI as an important feature of health and well-being in older adults. The purpose of this review is to summarize and evaluate existing research in this innovative area of scholarship, identifying the potential benefits and risks of both pet ownership and animals in therapeutic settings for older adults. We will also identify recommendations for future research and applications in this developing area of scholarship.

## Introduction

Pet ownership is prevalent in the United States; 65% of households have at least one pet (American Pet Products Association, 2016). Animals are increasingly incorporated into therapeutic settings targeting improved mental and physical health (e.g., Fine, 2015). The science of Human–Animal Interaction (HAI) seeks to understand how our relationships with animals can influence both human and animal health, and has grown considerably in recent years (McCune et al., 2014).

One particular area for continued growth is the role of HAI in healthy aging, given the potential for HAI as a key component of understanding health and well-being in older adults. Initial work in this area has demonstrated promising outcomes. The purpose of this paper is to describe existing research on HAI and older adults, and identify avenues for future research in this developing area of scholarship. Other reviews of HAI and aging exist, but are more limited in scope (e.g., limited to pet ownership; McNicholas, 2014), pathology (e.g., depression; Virués-Ortega et al., 2012), or are restricted to one type of HAI (e.g., Virués-Ortega et al., 2012; Bernabei et al., 2013) or one population (e.g., persons with dementia; Filan and Llewellyn-Jones, 2006). In this overview, we will draw on these reviews, along with edited volumes and original research, in order to frame the current state of the field with regards to HAI in healthy aging. As with much HAI research, the preponderance of the findings discussed herein is from work undertaken in Western countries.

As will become apparent the theme of the discussion below is that the impact of animals on healthy human aging is complicated and fraught with challenges. In this paper, we provide an overview of the state of this research, briefly describe the challenges, and make recommendations for future researchers.

## Overview of HAI Research

In exploring how HAI can impact older adults, it is important to understand the contexts in which they interact with animals, both as pets and in therapeutic situations. Pet ownership is one of the most common and sustained forms of interaction; pets may live in the home (e.g., cats and dogs) or outside (e.g., horses). An examination of the Health and Retirement Study data revealed that just over half of adults over the age of 50 years have at least one pet (author calculations using 2012 data). In addition, animal-assisted activities (AAAs) and therapy (AAT) are becoming increasingly popular for older adults. Animal-assisted activities involve informal activities such as animal visitation programs, whereas AAT is defined as “a goal oriented, planned, and structured therapeutic intervention directed and/or delivered by health, education, or human service professionals” (Jegatheesan et al., 2015, p. 416). As AAA/T programs designed for older adults become more commonplace, it is increasingly important to build an evidence base about the effectiveness of these practices. Existing research in both pet ownership and AAA/T has demonstrated promising findings for a variety of outcomes of health and well-being for older adults. However, this evidence base is not strong in all areas of health. We begin by presenting existing evidence for specific areas of health and well-being, with a focus on older adults, and then discuss the associated challenges.

### Cardiovascular Health

Some of the strongest research exploring HAI and health outcomes, though not specifically focused on older adults, has focused on the link between HAI and cardiovascular health and adaptive stress responses. Pet ownership has been associated with lower blood pressure, lower heart rate, and faster recovery during mental stress (Allen et al., 2001, age not specified; Allen et al., 2002, adults of all ages). Friedmann et al. (2013) demonstrated a link between pet ownership and cardiovascular health, both as a predictor of long-term survival in patients (ages 33–84 years) who had experienced a myocardial infarction (Friedmann et al., 2011), and in improving ambulatory blood pressure in hypertensive older adults. Aiba et al. (2012, participant ages: *M* = 69, *SD* = 8) showed pet ownership to be an independent predictor of cardiac autonomic imbalance for patients with conditions such as diabetes, hypertension, or hyperlipidemia. In fact, the American Heart Association (AHA) issued a scientific statement suggesting that pet ownership (particularly dogs) may reduce the risk of cardiovascular disease (Levine et al., 2013). Specifically, the AHA statement identified and summarized the 31 most relevant studies and concluded that there were varying levels of evidence (depending on outcome) for a link between pet ownership and improved outcomes regarding systemic hypertension, hyperlipidemia, physical activity, obesity, autonomic function, cardiovascular reactivity, and survival for individuals with cardiovascular disease. In addition to pet ownership, animal-assisted physical health interventions (primarily dog walking) have been associated with increasing cardiac functioning for older adults (Motooka et al., 2006) compared to when the same individuals were not walking with a dog, and with retaining functionality after stroke (Rondeau et al., 2010). However, more research is needed focusing specifically on older adult populations.

### Depression and Anxiety

Much HAI research has focused on the role of animals in mitigating mental health disorders such as depression/anxiety. Animal-assisted activities and therapy has been associated with reductions in depression symptoms for a variety of populations (Souter and Miller, 2007), with moderate effect sizes (Virués-Ortega et al., 2012). These effects are particularly apparent with populations in elder-care institutions and assisted-living facilities, such as older adults with no cognitive impairment (Colombo et al., 2006), with dementia (Travers et al., 2013), requiring the use of wheelchairs or walkers (Le Roux and Kemp, 2009), and with mental illness (Moretti et al., 2011). Similarly, AAA/T has been shown to reduce anxiety in patients with Alzheimer’s disease (Mossello et al., 2011), those hospitalized with heart failure (Cole et al., 2007), and residents of long-term care facilities (Le Roux and Kemp, 2009).

In addition to evidence of the therapeutic effects of AAA/T, some findings have indicated a link between pet ownership and reduced depression for older adults (e.g., Garrity et al., 1989); the emotional bond between older adults and their pets seems to be a particularly important component of that relationship (Peretti, 1990). However, several studies have not found differences in levels of depression between pet owners and non-pet owners (Miller and Lago, 1990; Branson et al., 2016), or have found an increase in depressive symptoms after the loss of a pet (Chan et al., 2012). More research is needed in this area to clarify the impact of pets on depressive symptoms.

### Loneliness and Social Support

Human–animal interaction may provide social support for older adults, thus reducing the risk of loneliness. Animal-assisted activity has been leveraged as an intervention to improve social functioning in older psychiatric (Haughie et al., 1992) and dementia patients (Sellers, 2005; Filan and Llewellyn-Jones, 2006; Marx et al., 2010). In addition, AAA can contribute to reducing loneliness, and improving quality of life, mood, and social interaction (Banks and Banks, 2002; Vrbanac et al., 2013) in long-term and elder-care facilities.

Pet ownership has been hypothesized to have similar beneficial effects. Older pet owners have been found to be less likely to report loneliness (Stanley et al., 2013). Pets may act as catalysts for human social interactions (McNicholas and Collis, 2000), thereby fostering a sense of community (Wood et al., 2007). In a study of adults 50+ years of age, Toohey et al. (2013) found that frequent dog walkers were more likely to report a high sense of community, in addition to the benefits of increased physical activity. In addition, for older adults high attachment to a pet has been associated with lower levels of loneliness (age 55–84 years; Krause-Parello and Gulick, 2013) and appears to mediate the relationship between loneliness and general health for older women (age 55–84 years; Krause-Parello, 2008), perhaps providing a “buffering” effect. However, there are also mixed findings in this area; Gilbey et al. (2007) found no difference in loneliness between pet owners and non-pet owners in a sample of adults 18 years and over. Since these studies have been correlative, it cannot be ruled out that lonely individuals are more likely to get pets; therefore, future work assessing causality is necessary (Pikhartova et al., 2014).

### Physical Activity and Falls

In addition to mental health and well-being outcomes, pet owners (and dog owners in particular) are often more likely to be physically active. Older adult dog owners are significantly more likely to walk more than non-pet owners, and have higher levels of physical activity (Dembicki and Anderson, 1996; Thorpe et al., 2006; Toohey et al., 2013; Curl et al., 2016). In addition, one study found less deterioration in ability to perform activities of daily living for older adult pet owners compared to non-pet owners (age 65+; Raina et al., 1999). Recently, a study using the ActivPal accelerometer showed that independent living older adult dog owners spent more time walking, walked for more minutes at a moderate cadence, and spent less time sitting compared to their non-dog-owning matched counterparts (Dall et al., 2017). Within the context of AAA, assisted-living residents who walked with a dog walked farther compared to walking without a dog (Herbert and Greene, 2001).

In addition to the physical health benefits of HAI, it is also important to note that animals may be associated with increased risk. Falls are a major factor influencing mortality and morbidity for aging adults (Ambrose et al., 2013). Dogs and cats may be associated with fall risk, and fall injuries related to pets are highest for people 75 years of age and older (Stevens et al., 2010). In addition, dogs can be a hazard for injuries related to leash-walking (Willmott et al., 2012) and both cats and dogs can be linked to scratches and bites. Although the risk of disease spreading between animals and people (zoonotic disease transmission) is low (e.g., Lowden et al., 2015), it is important to recognize that older adults likely represent a vulnerable population. Additional research is needed to explore the risk/benefit ratio of HAI for older adults, and to establish best practices for ensuring safety and supporting pet ownership, particularly in physical activity interventions involving dog walking.

### Quality of Life and Life Satisfaction

Quality of life is a key issue for aging adults as they move through life transitions, and interacting with animals may be a way of mitigating some of the associated stressors (Steed and Smith, 2002). Pet ownership has been linked to smaller decreases in life satisfaction after retirement (Norris et al., 1999). Interestingly, one study found that caregivers of a spouse with dementia reported higher attachment to their pets after onset of their spouse’s dementia (Connell et al., 2007). Animal-assisted activities also have been associated with increased life satisfaction and decreased depression in older adults, including those both with and without dementia or cognitive deficits (Steed and Smith, 2002; Colombo et al., 2006; Olsen et al., 2016).

## Considerations/Challenges for HAI Research

Research on HAI has been plagued by methodological shortcomings which call into question the strength of the evidence base. The preponderance of the published research lacks the use of standardized measures for concepts such as pet bonding and for outcomes (e.g., loneliness), relies on small sample sizes, and involves short-term examination of effects (lack of longitudinal studies). The animals involved and the human interaction with animals are frequently not well described and rarely is pet ownership history or degree of involvement with pets included when describing the sample and methodology (for a complete discussion see Kazdin, 2015). In research involving AAA/T, many studies do not employ randomized controlled trials or other rigorous control or comparison procedures such as use of appropriate covariate procedures in data analysis. As HAI researchers begin to more seriously investigate the impact of animals on older adults, it is critical that they carefully examine their planned research approaches and simply put, raise the bar on quality. Further, as with any field of investigation, there is a positive publication bias – typically journals are more likely to accept for publication manuscripts that report significant findings (Herzog, 2015). This bias is exacerbated in HAI research because often researchers form hypotheses that test for associated benefits, rather than posing more objective questions, and fail to consider that HAI could have negative effects (Stevens et al., 2010).

In addition, there are certain methodological challenges specific to the nature of HAI. For example, it is nearly impossible to conduct double-blind studies involving animals – participants are aware that the animal is present. Finally, in examining the impact of pet ownership, there is a fundamental confound involving causality that is difficult to avoid. In most studies of pet ownership, due to selection effects, it is difficult to ascertain if pet ownership is the cause of certain health outcomes – do pets make people healthier or do healthier people choose to own pets? One could address this by randomly assigning pet ownership to participants (e.g., Allen et al., 2001), but because pet type and ownership are personal choices with long-term implications, few participants are willing to be assigned to a particular pet ownership status. Another approach is to utilize large-scale, longitudinal datasets that allow for the use of comprehensive control variables at the analysis phase to identify some of the factors predicting pet ownership; however, as elegant as this approach may be, it is still not possible to completely determine causality.

## Considerations/Challenges for Aging Research

It is important to understand the challenges of integrating HAI research with aging research. When conducting research with older adults, there are a number of design and implementation considerations. For example, researchers must decide how to define the lower age limit to be considered an “older adult.” In the United States, age 65 years is used for federal statistical purposes (e.g., Administration on Aging [AoA], 2016), while the United Nations (2015) generally uses 60 years as the cut-off age for “older adulthood.” Alternatively, researchers may define older adulthood functionally, in terms of being retired or living in a retirement community. In addition, researchers often create age groupings of young–old, old–old, and oldest–old (Garfein and Herzog, 1995), and may vary in how they define such groups (e.g., Garrity et al., 1989; Toohey et al., 2013).

Aside from the characteristic of age, there is a great deal of heterogeneity among the older adult population, including gender, marital status, living arrangements and housing, racial and ethnic composition, geographic distribution, income and wealth, employment, education, health, and health care (Administration on Aging [AoA], 2016), all of which may be important variables in understanding HAI in older adults (e.g., living alone; Stanley et al., 2013). Therefore, researchers should consider how to recruit study participants to reflect these characteristics, and take care when making statements about the generalizability of findings (Nesselroade and Labouvie, 1985).

Despite the heterogeneity in health status, there are general patterns of age-related declines in health, which can impact research participation: normal age-related declines in hearing, vision, cognition, and dexterity can create difficulties for older research participants (Weil, 2015). Age-related health changes can also pose mobility and transportation problems in traveling to research sites if a participant is in a wheelchair and/or cannot drive. These changes might also impact an individual’s ability to participate in HAI, particularly in activities such as dog walking (Toohey et al., 2013).

Experimental and longitudinal researchers face additional challenges: older participants often drop out of research studies after moving, when experiencing illness and/or hospitalization, because of cognitive decline, or because they simply decide to stop participating (García-Peña et al., 2015a). Attrition can be reduced by allowing someone else (a “proxy”) to provide responses, but this can be problematic when trying to examine change (Weil, 2015). Other options include multiple follow-up attempts, reducing the length of time between observations, and use of other modes of contact (e.g., telephone and internet). Older participants are also at an increased risk of mortality.

Normal age-related changes and increased risk for health problems must also be considered (García-Peña et al., 2015b). For some conditions, it might be more realistic to test whether an intervention is effective in slowing the rate of health decline, rather than resulting in improvements. Similarly, in longitudinal studies, the measurement approaches may change over time. While changes may reflect methodological improvements and/or simplification to reduce burden, these changes also can create problems when studying change over time. Researchers should carefully consider the amount and nature of changes in measurement when designing studies and publishing findings. Secondary data sources may also not include all relevant measures for a research topic, and/or questions may be asked in a way that reduces the measure’s psychometric properties or comparability to other research studies.

In aging-related studies, researchers are faced with the disentangling age, period, and cohort effects (Nesselroade and Labouvie, 1985). Age effects refer to chronological age-related changes, while period effects are the historical context and effects of a given timeframe (e.g., natural disaster). Cohort effects describe the way that age generations tend to act and behave. Disentangling these effects can be difficult if not impossible, and researchers should carefully consider which effects are of greatest interest. Researchers should be wary of attributing differences to age when they could be explained by period or cohort effects. A related consideration is that of continuity versus change – that is, differences between individuals could have existed for years and extend into older adulthood, or they could have emerged as part of the aging process.

## Recommendations for Future Research

Of critical importance to future research in the intersection of HAI and gerontology is high quality methodology, as has been called for in other areas of HAI (McCune et al., 2014). This nascent research focus will benefit from the broad spectrum of investigative approaches from qualitative and descriptive, to large-scale longitudinal studies, to the gold standard of establishing causation: the randomized controlled trial. In all cases, the key is to implement the best design to appropriately address the question under investigation. The use of high quality, rigorous methodologies is critical to establishing a solid foundational evidence base.

It is important for researchers to be comprehensive in presenting the relevant background research. In addition to presenting findings that are published in both HAI and gerontology journals, researchers in both fields also frequently publish in a wide variety of other disciplines (e.g., psychology, sociology, and anthropology). This breadth of publication outlets may make it challenging to consolidate and organize all the relevant previous research. We recommend that researchers develop interdisciplinary teams that bring the relevant HAI and gerontology expertise to projects.

Another important aspect of building an evidence base is the use of theory to guide research. There is a need to identify the important issues in both fields and which theories best provide a framework to guide their investigation. For example, attachment theory may help us to better understand the bond that older adults form with their pets, and social support theory may help us to understand how and for whom pets may serve as alternate support figures when older adults lose key support figures such as spouses (e.g., Beetz, 2017).

There are many areas within HAI and aging ripe for future research. For example, within the context of pet ownership, what factors are important to consider when trying to help “match” people with pets? Are there contraindications for pet ownership, and what barriers to pet ownership might exist that are unique to the older adult population? In AAA/T, there is a need for additional rigorous, longitudinal research to assess the clinical effects of these interventions. For example, is AAA/T helpful in managing or treating dementia, or sustaining cognition in older age? And are there specific barriers or potential issues related to using AAA/AAT with older patients – for people and for animals?

Investigating HAI in older populations provides opportunities to examine how the impact of HAI may vary developmentally and across other demographic characteristics. Research can also examine the impact of HAI on a variety of outcomes (e.g., physical and mental health) and unearth the mechanisms by which these effects operate, ultimately better informing the ways in which pets may impact the lives of our rapidly growing older adult population.

## Conclusion

Although it is clear that the impact of animals on healthy human aging is complicated and fraught with challenges, we believe this exciting research area is ripe for growth. While initial findings in many areas of health and well-being are promising, there are numerous opportunities for improving the breadth, depth, and quality of the research on HAI and human aging. As such, the field is at an exciting and critical turning point, with the potential for significant growth in our understanding of how animals can impact older adults in ways that may support meaningful changes to policy and practice in the future.

## Author Contributions

All authors listed have made a substantial, direct and intellectual contribution to the work, and approved it for publication.

## Conflict of Interest Statement

The authors declare that the research was conducted in the absence of any commercial or financial relationships that could be construed as a potential conflict of interest.
